# Hospital and Institutional News

**Published:** 1918-05-11

**Authors:** 


### HOSPITAL AND INSTITUTIONAL NEWS.

### OUR BROTHERS OF THE UNITED STATES.

On another page will be found an interesting article dealing with the relations of Army and Navy patients to civil hospitals in America. It cannot fail to be of interest to ourselves, and shows the unity and co-operation which the war is bringing about and its quickening influence for brotherhood, mutual counsel, and help. In this connection we are able to announce the interesting fact that an American, Dr. S. S. Goldwater, an expert in hospital construction, has been invited by the authorities in Japan to develop suitable plans for St. Luke's Hospital, Tokio. It is interesting for more reasons than one that the Japanese bought the site before consulting the expert. It is an irregular triangular plot, wide at the north, and narrowing almost to a point at the south, and not very widely extended in any direction. The difficulty of developing a satisfactory plan on such a plot must necessarily be great. It will mean hard thinking on the part of Dr. Goldwater, who is an indefatigable worker, and will be, no doubt, pleased with his problem for that, amongst other reasons. We have had some grateful testimony from the United States. The chairman of the War Service Committee of the American Hospital Association, of the Hospital Committee of the Council of National Defence, and of the Mayor's Committee on Hospital and Medical Facilities, New York City, writes to say that in his work as chairman he finds "the columns of The Hospital invaluable. From no other source has it been possible to obtain so much practical information concerning hospital arrangements abroad which have been necessitated by the war, or so much illuminating comment on the principles involved and the methods employed." John Delane held that a good editor should seldom write. Sharing Delane's view, this welcome and grateful testimony is published without comment.

### SPECIAL RATIONS SCALES FOR HOSPITALS.

On page 96 of our issue of May 4 we published an authoritative diet scale giving the rations for hospitals applicable to the patients and the professional staffs, including the nursing staff. The Ministry of Food, under the date of May 6, further intimates that, in addition to civil general hospitals and most special hospitals, the scale is applicable to Poor-Law infirmaries, to the sick wards of other establishments classed as institutions under the Meat Rationing Scheme, and, under certain conditions, to convalescent homes and nursing homes. In the case of hospitals entitled to a special institutional scale, the medical, surgical, and nursing staffs will receive the civil general hospitals scale, except that at sanatoria they will, if themselves tubercular, receive the same scale as the patients. Applications to be registered as entitled to a special institutional scale must be made to the Local Food Office. The same hospital may apply for more than one scale when it provides for naval and military patients, civilian patients, and professional staff, and tubercular patients in special wards, in addition to other residents and non-residents receiving ordinary rations as members of the general public.

### HOSPITALS AND THEIR MILK SUPPLY.

The results of the investigation concerning London hospital milk-supplies, recently carried out at the Lister Institute for the National Clean Milk Society, reveal no new facts and afford no ground for satisfaction. They only re-establish the belief that it is impracticable, indeed impossible, to obtain in London, under present conditions, pure and clean milk. Were good milk procurable the hospitals are clearly in a position to secure it. Yet of the milk delivered to twenty-one hospitals 10 per cent. of the samples revealed tubercle and every one contained organisms of the coli group, such as are usually derived, when found in milk, from manure. Although the samples appear to have been taken during the winter months, eight contained upwards of 1,000,000 bacteria per cubic centimetre, one showing the stupendous count of 250,000,000! We need not enter upon the consideration of the condition of such a milk after an all-night journey in summer-time without any provision for cooling. The National Clean Milk Society aims at breaking down the existing apathy regarding the purity of our milk-supplies and at eliminating such widely-prevalent practices as that of "pasteurising" stale milk with the object of keeping it fit to be passed as "new" milk to the consumer. It is to be remembered that this process, as used, has not for its object the destruction of tubercle, nor is it carried to a point at which it can be relied upon to do so. London milk has become in the main a "faked" trade-product, and is, when judged by the standard of pure new milk, a dangerous snare and delusion to the unwitting consumer.

### "EARLSMEAD" HOME OF RECOVERY.

The new branch of the Great Northern Central Hospital, the "Earlsmead" Home of Recovery in Hornsey Lane, has now been opened. The Mayor of Hornsey, Councillor Percy Fone Teychenne, J.P., presided. Lady Crosfield declared the Home open. Mr. H. J. Tennant, M.P., deputy chairman of the hospital, gave an outline of the hospital's progress, showing that since the outbreak of war the number of beds had been increased from 211 to 430 (including 25 at "Earlsmead"). On behalf of the hospital, he thanked Mr. E. G. Harrop for placing his private house at the service of the committee of management for the duration of the war. After a prayer by the Bishop of Willesden, a vote of thanks to Lady Crosfield was proposed by Mr. F. Hammond, F.R.I.B.A. (chairman of the Convalescent Homes Committee), whose appeal for voluntary collectors for the Home met with a satisfactory response. The Women's Theatre was responsible for an attractive programme, after which ration tea was provided by the Executive

Committee of the Ladies' Association, who took charge of the many gifts presented to the Home during the day.

### A HISTORICAL DOCUMENT.

IN connection with the attack on the hospital ship *Guildford Castle*, the Secretary of the Admiralty has issued a statement in which "it is now considered to be conclusively proved that the ship was attacked by a German submarine on the afternoon of March 10 and was struck by a torpedo, and that the ship had a narrow escape of being sunk." Happily the torpedo did not explode. The Secretary of the Admiralty goes on to quote an extract from the German official message sent through the wireless stations of that Government on April 24. In criticising the Admiralty official returns of shipping losses, the *Deutsche Tageszeitung* declares: "Lloyd George and Geddes falsify the losses of ships plying in the military zone [? ignoring] so-called naval losses, auxiliary cruisers, guard ships, *hospital ships*, and very probably also troop transports and munition steamers—that is to say, precisely that shipping space *which is particularly exposed to and attacked by the U boats*." In the welter of falsehoods and mystifications in which warfare is carried on, anything approaching direct evidence or admission is welcome. The documents of the present war make such evidence not only welcome, but precious by its rarity.

### KING EDWARD MEMORIAL HOSPITAL, BIRMINGHAM

FOR the building and equipment of the new King Edward Memorial Hospital for Children, Birmingham, an appeal for £25,000 is to be issued. In its fourth year the hospital has received 748 in-patients, but is still hampered by the distance at which the out-patient department is situated. The death-rate, by the way, was 18.05 per cent. The hospital can put forward two arguments on behalf of the proposed appeal; the one immediate, the other remote. The immediate point is that if new money enable it to open another ward, the other hospitals of Birmingham will be relieved of some of their child patients, and the waiting-lists at Birmingham are far too long. The remote argument is that when the King Edward Memorial is in full working order it will be the largest children's hospital in the Midlands. More beds are badly wanted, and children's hospitals are still a popular cause. The new effort, however, will not be made under Mr. W. E. Pearson's leadership, for he is resigning the chairmanship of the committee on the ground that he has too much business to attend to. Mr. Cheshire, the president, is an enthusiast, however, and no doubt will make every effort to succeed in an hitherto uphill task.

### THE ANATOMICAL SOCIETY OF GREAT BRITAIN AND IRELAND.

THE Anatomical Society of Great Britain and Ireland, having received and unanimously adopted the report presented to it by its Committee on Nomenclature, resolved, without a dissentient vote, that "it sees no reason for departing from the use of the old nomenclature as the recognised medium of description for employment in anatomical text-books and departments, or by medical men in general; on the other hand, it thinks that there are very good reasons to be urged against the adoption of any other nomenclature for this purpose."

### VACCINATION OF CHILDREN IN POOR-LAW INSTITUTIONS.

A SUGGESTION made by Dr. Lionel Baly, medical superintendent of Lambeth Infirmary, has been strongly opposed by the Southwark Board of Guardians, whose patients are now being treated by the Lambeth authorities. Dr. Baly wrote to the clerk requesting that the relieving officers should be asked to call special attention of all parents of children or patients admitted to the infirmary that in case of necessity they would be vaccinated unless a certificate of conscientious objection was produced. The clerk stated that immediately on receiving this letter he had written to Dr. Baly informing him that he had no legal power to vaccinate any child without first of all receiving the consent of the parents, and his action was approved by the Guardians. This is the legal position, unless the Lambeth Board of Guardians declines to receive patients who fail to give an undertaking that they will raise no objection to vaccination if it is deemed necessary. The Southwark Board of Guardians have always been opposed to compulsory vaccination, maintaining that every person had the right to his or her own opinions on the matter, and this policy they have pursued with regard to Southwark patients sent to other institutions outside their jurisdiction.

### THE BEGINNING OF THE SATURDAY FUND.

SYMPATHETIC allusion was made by the Lord Mayor at the annual meeting of the Hospital Saturday Fund to the death of Sir James Whitehead, the first Lord Mayor president of the Fund. Sir James, the Lord Mayor remarked, had been a very great benefactor of the Saturday Fund, having instituted the Penny-a-Week collection. From that small seed sown many years ago the Fund with its "fruitful branches" had grown. Sir T. Vezey Strong observed that the total receipts for 1917 had reached the record amount of £59,228, as compared with £46,756 in 1915. An address on the "Food Problem" was delivered by Professor Sterling.

### A BEDROOM BAZAAR.

AN instance of the value of a patient's gratitude has again occurred in connection with the Royal Victoria and West Hants Hospital, Bournemouth. For the third time Miss Arter, an old patient of this institution, has organised a bazaar in aid of its funds. Miss Arter is an invalid who is confined to her bed, and the novel idea of a bazaar in her bed-sitting room has been once more successful. The bazaar was opened by the Dowager Countess of Malmesbury, a great friend of the hospital, and resulted in the sum of over £55 being added to the funds of the institution. When it is remembered the bazaar was organised entirely by Miss Arter from her bed, and that all the articles sold were the work of her friends and herself, including other old patients of the hospital, it will be realised with what enthusiasm and genuine gratitude she has worked. The hospital has received a total of over £100 from this source.

### THE WOMAN HOUSE SURGEON.

It was lately stated by Judge Greenwell that the experiment of having a woman surgeon at Durham County Hospital was proving most satisfactory. Even to-day such appointments are regarded as experimental, and therefore every evidence on either side is worth attention. The increasing numerical proportion of women in the medical profession is seen by analysing the two lists lately issued by the University of London of the second examination for medical degrees, Parts 1 and 2. Of the 162 successful candidates 100 were women and 62 men. The majority of women came from the Royal Free Hospital Medical School for Women, and this evidence of its activity and importance coincides with the ambitious policy which is now engaging the attention of the hospital's committee. These numbers are also significant when we remember the hospitals which have come to depend during the war on the assistance of unqualified men.

### A REQUEST AND A GIFT TO PORTSMOUTH HOSPITAL.

In consequence of a communication from the Ministry of Pensions as to the relation of the hospital accommodation to the needs of the neighbourhood, a special committee of the Portsmouth and Gosport Hospital has recorded its opinion that an additional ward block will be required after the war, and that voluntary funds are unlikely to be sufficient to pay for it. The matter is complex because, as Mr. T. H. F. Lapthorn, the chairman, has explained, the present laundry would have to be scrapped before the new ward was built, since the old one could not deal with the increased work that would follow. Moreover, a new kitchen and a new mortuary would also be required, but Mr. Lapthorn thinks that there may be some chance of a subsidy. Besides having treated a number of shipwrecked sailors, applications for the admission of discharged men from both Services are received. The money received for the treatment of cases of venereal disease helps to set off the rising cost of provisions, and a curious donation has been received from the British Church Establishment, Canton, China. This represented sums taken at a piano recital last January at Shameen, and at an organ recital two days later. The latter was held in aid of a fund to endow a bed in the Royal Hospital, Porstmouth, to be called the Canton Bed, for the children of sailors and marines, in memory of the officers and men of the Allied gunboats at various times stationed at the Canton Delta.

### A NEGLECTED SOURCE OF INCOME.

In spite of the success of the recent matinée at the Palladium, the new Chelsea Hospital for Women is still in debt to the extent of £11,000, and there is an overdraft of £5,000 on the current account. The first floor of the hospital has been taken by the War Office for wounded officers. It is true that annual subscriptions show a slight increase, but much remains to be done. London is so big a city that the London hospitals look rather to the great funds and to special appeals than to the organisation on which the provincial hospitals have come to depend for the principal source of their income. We should have thought that it was not impossible to organise sectional subscriptions in London also, and that a woman's hospital was in a favourable position to do so now that women are grouped in so many professions and enterprises of their own.

### A DOUBLE GIFT.

For the second time Mr. F. W. Pascoe Rutter has given a house for use as a military hospital. Rather more than twelve months ago he presented his house, Windy Knowe, at Blundellsands, near Liverpool, to the military authorities for the use of the wounded. His latest gift is Dawpool, Thurstaston, Cheshire, which he has handed over as an orthopædic hospital for officers. The house, which is affiliated to the Alderley Military Hospital, Liverpool, has accommodation for seventy officers.

### LEGACIES AND MAINTENANCE.

The General Hospital, Walsall, is using the opportunity provided by the two recent legacies of £4,000 from Mr. James Mason, of Melbourne, and £20,000 from Mr. Joseph Taylor to clear the way for further extensions by paying off the existing debt. The first legacy, which is ear-marked for a James Mason Ward, is not big enough to build and maintain the ward of twenty beds which is required. The second will help to provide for its maintenance, but on the success of the forthcoming appeal depends the liquidation of the debt so that the hospital committee may be set free to use these two legacies in the way which the testators hoped for.

### STAFF CHANGES AND ANNUITIES AT NOTTINGHAM.

The resignation of Miss Knight from the matronship of Nottingham General Hospital has occurred after twenty-five years of work, and the retiring president, Mr. W. G. Player, declared of her that she had worn herself out in their service. Mr. Player is succeeded in the presidency by Sir Charles Seely, M.P., who will doubtless carry on the tradition set by the late Sir Charles Seely, a benefactor of the hospital second to none. Mr. Player has marked his retirement by the gift of £1,000 to endow a bed in the name of his wife and his eldest daughter, who he was proud to say has worn a nurse's uniform for two years. We are glad to note the testimonial to Miss Knight will take the form of an annuity. The hospital has no pension fund for the nursing staff, though years ago Mr. Bradshaw gave the sum of £500 as a nucleus. This is where the Royal National Pension Fund for Nurses comes in.

### DOCTORS' CERTIFICATES.

A workman has been fined £2 by a local Munitions Tribunal in Glasgow in connection with a doctor's certificate. The man, who had been released from the Army, submitted a doctor's certificate stating that he was unfit to work on the day on which it was granted. The certificate, which made no statement of the man's illness, was described by the chairman as utterly useless for the purposes of the Tribunal. The doctor declared that the nature of the illness was only stated when the certificate declared unfitness for more than one day. In these days, when demands are general, doctors are placed in a difficult position. Certificates inevitably result from the mass of existing regulations, and yet it is more difficult than ever for medical men to grant them without laying themselves open to suspicion of carelessness or partiality.

### THE HEALTH OF LONDON SCHOOL CHILDREN.

The war has, we are told, led to a cheapening of the value of human life, and the constant spectacle of suffering and death upon the battlefield might well lead to a disregard at home of those forms of social endeavour that have for their end the betterment of mankind. To how small an extent these gloomy prognostications are justified is seen in the recently published report of the Children's Care Committee of the L.C.C., based on the examination of 200,000 school children. The health of London's children is actually better now than it was in 1913; less than half the number of children entering the schools are found to be in a poorly nourished condition than in the year before the war. Moreover, there has been an all-round improvement in health, first noted in 1916 and fully maintained during 1917. Having in mind the loose talking indulged in relative to the increase of alcoholism amongst the wives of soldiers and the consequent neglect of homes and children, these figures are indeed remarkable. That the poor of to-day should be able to feed their children better than in the "piping" times of peace is a comment on social conditions that needs no elaboration.

### THE WAR AND INFANT MORTALITY.

Only recently we were congratulating ourselves upon the remarkable decrease in infant mortality during 1916, when the average figure for the country was 91 per thousand, the lowest ever recorded. But the provisional returns of the Registrar-General which have lately been issued effectively remove any complacency we may have felt. Not only has the infant mortality rate risen 6 per thousand in country districts and in the ninety-six large towns including London, but in London itself there has been a rise of 16 per thousand. The *Lancet*, commenting upon this remarkable increase, attributes it to the cumulative action of prolonged mental strain upon the nursing mother. Welfare centres in districts which have suffered much from the devastation of air raids state there has been a large diminution in the number of infants solely breast-fed up to the time of weaning, and this, coupled with the enforced sleeplessness and general irregularity of habits consequent upon long hours of "taking cover," cannot have failed to have a highly prejudicial effect upon the health of the tender infant. Moreover, the close gathering together of large bodies of people in Tubes and shelters is highly favourable to the spread of contagious disorders of all kinds. One is inclined, indeed, to wonder whether the risks so run are not far greater than are to be feared from a bomb or chance fragment of shrapnel.

### MUNICIPALISING THE NURSERIES.

The Health Committee of the Glasgow Corporation has decided to take over the institutions of the Glasgow Day Nurseries' Association and maintain the nurseries as part of the Child Welfare Scheme. The annual cost thereby assumed by the municipality is about £1,200, whilst the outstanding debts, about £200, of the Association will be paid by the Corporation. The staff now employed will be taken over on the same terms as they now hold office, and when the transference is complete the Association will be wound up. The Corporation, by the way, is considering the establishment of a country home for children, there being a proposal to utilise Balloch Castle for the accommodation of delicate children up to five years of age.

### THIS WEEK'S DRUG MARKET.

The difficulty of obtaining supplies of various drugs and medicinal chemicals on the open market is becoming more pronounced, and suppliers are obliged to continue to ration their customers. This does not necessarily mean an intrinsic scarcity, for it must be remembered that the requirements of the Medical Services of the Navy and Army have to receive prompt consideration. Ethers are not readily procurable in the ordinary way of business. Supplies of carbonate of magnesia are being strictly rationed. The English makers of quinine can only supply a small proportion of the demands made on them. The demand for citric acid is brisker, and it seems probable that prices will again advance. Bromides are quieter, and prices are not quite so firmly maintained, except for the ammonia salt, which is scarce and dearer. Sodium phosphate is much dearer. Eucalyptus oil is in better supply, and as the demand is rather slow prices are not so firmly maintained; some of the recent arrivals of oil are of inferior quality. Quotations for aloin and podophyllin have been advanced. Ergot of rye continues to move upwards in price. Tannic acid and gallic acid are dearer. Sarsaparilla is not so scarce. In the absence of adequate supplies of British-made saccharin a good business continues to be done in the imported product at advancing rates.

### TO OUR READERS.

**Contributions are specially invited from any of our readers to these columns. They should deal with topical subjects and news. They must be authenticated for the information of the Editor only. The minimum payment if published is 5s. There is no hard-and-fast rule as to space, but notes of about twenty lines in length are preferred.**

